# Supplementary material for: Predicting Mortality Using Machine Learning Algorithms in Patients Who Require Renal Replacement Therapy in the Critical Care Unit
Source: J Clin Med. 2022 Sep 8;11(18):5289. doi: 10.3390/jcm11185289 (PMC9500742; doi:10.3390/jcm11185289)

Supplementary Materials

Table S1. ICD-9 diagnosis codes used to identify acute kidney injury, transplant history, and comorbidities.

Diagnosis	ICD-9 diagnosis codes
Acute kidney injury	584.x, 634.3, 635.3, 636.3, 637.3, 638.3, 639.3, 669.3, 958.5
Diabetes mellitus (DM)	249', '250'
Hypertension (HTN)	'401', '402', '403', '404', '405'
Chronic kidney disease (CKD)	'585', '586'
Liver cirrhosis (LC)	'571.2', '571.5', '571.6', '572.2', '572.3', '572.4', '572.8'
Cancer	Between '196.0' and '199.1', between '209.70' and '209.75', '209.79', '789.51' / Metastatic cancer /
	Between '200.00' and '202.38', between '202.50'

	and '203.01', between '203.02' and '203.82', '238.6', '273.3' / Lymphoma /
	Between '140.0 ' and '172.9', between '174.0 ', between '258.01' and '258.03', between '209.00' and '209.24', between '209.25' and '209.30', '175.9', '179', '195.8', '209.30', '209.36' / Solid tumor without metastasis /
CKD stages 4 and 5	585.4, 585.5
End-stage kidney disease	585.6
Transplant	V42, 996.8

Table S2. Percentage of missing data in MIMIC and eICU datasets

Database	eICU	MIMIC
BUN (mg/dL)	0.26%	0%
FiO2 (%)	21.98%	25.50%
HCO3 (mmol/L)	2.42%	0.17%
Hgb (mg/dL)	4.14%	0.88%
O2 Sat (%)	2.76%	29.55%
WBC count (x1000/µL)	5.00%	0.71%
Anion gap (mmol/L)	20.03%	1.24%
Calcium (mg/dL)	2.03%	6.28%
Chloride (mmol/L)	0.26%	0.08%
Creatinine (mg/dL)	0%	0%
Glucose (mg/dL)	0.08%	0.08%
Platelet count (x1000/µL)	5.18%	0.44%
Potassium (mmol/L)	0.17%	0%

Sodium (mmol/L)	0.08%	0%
GCS score	11.35%	1.77%
MAP (mmHg)	0.58%	0.44%
HR (beats per minute)	0.25%	0.17%
RR (breaths per minute)	1.16%	0.26%

Table S3. Baseline characteristics of the patients requiring renal replacement therapy in the MIMIC dataset.

Variables	Survival	Death	*P* value
Number of patients	645	484	
**Demographics**			
Age, years	61.9 ± 15.9	64.4 ± 15.3	0.008
Male sex, %	59.7%	65.1%	0.074
Black race, %	14.0%	6.4%	<0.001
**Comorbidities, (%)**			
Diabetes mellitus	38.6%	31.4%	0.015
Hypertension	55.0%	49.0%	0.050
CHF	43.1%	40.3%	0.375
CKD	20.0%	17.1%	0.256
Malignancy	8.8%	12.4%	0.065
Liver Cirrhosis	12.7%	28.1%	<0.001
Days of ICU stay before RRT initiation	1.2 (0.4-3.4)	1.9 (0.7-4.9)	<0.001*

Diuretics, %	16.4%	19.6%	0.190
Vasopressor, %	23.3%	43.8%	<0.001
Mechanical ventilation, %	56.6%	78.3%	<0.001
**Laboratory variables**			
BUN (mg/dL)	65.7 ± 39.2	69.1 ± 38.7	0.154
FiO2 (%)	44.2 ± 27.1	47.7 ± 26.7	0.029
HCO3 (mmol/L)	21.0 (17-24.5)	19.0 (15.9-22.0)	<0.001*
Hgb (mg/dL)	10.2 ± 1.9	10.1 ± 1.5	0.228
O2 Sat (%)	95.3 (90.4-97.5)	94.5 (88.0-96.7)	<0.001*
WBC count (×1000/μL)	11.9 (8.4-17.4)	13.5 (8.8-18.4)	0.008*
Anion gap (mmol/L)	18.5 (16.0-22.0)	20.0 (17.3-23.8)	<0.001*
Calcium (mg/dL)	8.4 ± 1.1	8.4 ± 1.2	0.817
Creatinine (mg/dL)	4.4 (2.7-6.4)	3.7 (2.4-5.0)	<0.001*
Glucose (mg/dL)	143.8 ± 77.4	138.4 ± 57.6	0.198
Platelet count (×1000/μL)	181.0 (107.4-266.0)	117.5 (72.0-191.4)	<0.001*

Potassium (mmol/L)	4.6 ± 0.9	4.7 ± 0.8	0.753
Sodium (mmol/L)	137 (134.0-140.0)	137.1 (133.5-141.5)	0.225*
GCS score	14.0 (9.0-15.0)	10.0 (5.8-15.0)	<0.001*
MAP (mmHg)	75.0 (68.3-85.4)	69.9 (64.0-77.6)	<0.001*
HR (beats per minute)	86.0 (74.2-98.6)	89.5(76.4-103.5)	0.001*
RR (breaths per minute)	19.3 (16.2-23.0)	21.2 (17.9-25.8)	<0.001*

Data are expressed as n (%) for categorical data and as mean ± standard deviation or median (interquartile range) for continuous data.

* Mann-Whitney U test

Table S4. Baseline characteristics of the patients requiring renal replacement therapy in the eICU dataset

Variables	Survival	Death	*P* value
Number of patients	1531	752	
**Demographics**			
Age, years	63.0 (52.0-72.0)	66.0 (57.0-75.0)	<0.001*
Male sex, %	59.0%	57.2%	0.438
Black race, %	14.7%	10.2%	<0.001
**Comorbidities, (%)**			
Diabetes mellitus	15.3%	13.2%	0.199
Hypertension	14.0%	9.4%	0.002
CHF	16.7%	17.3%	0.750
CKD	17.7%	14.4%	0.051
Malignancy	4.3%	6.1%	0.076
Liver Cirrhosis	3.7%	10.6%	<0.001
Days of ICU stay before RRT initiation	0.9 (0.1-2.8)	1.6 (0.5-3.6)	<0.001*

Diuretics, %	11.8%	12.4%	0.724
Vasopressor, %	28.7%	60.5%	<0.001
Mechanical ventilation, %	68.6%	91.9%	<0.001
**Laboratory variables**			
BUN (mg/dL)	64.4 ± 38.4	58.7 ± 33.1	0.028
FiO2 (%)	40.0 (28.0-60.0)	60.0 (40.0-85.0)	<0.001*
HCO3 (mmol/L)	21.9 (17.2-25.0)	19.6 (15.8-23.2)	<0.001*
Hgb (mg/dL)	9.7 ± 2.2	9.8 ± 2.1	0.625
O2 Sat (%)	96.9 (94.7-98.6)	96.0 (92.7-98.0)	<0.001*
WBC count (×1000/µL)	12.4 (8.6-17.4)	14.0 (9.6-20.5)	<0.001*
Anion gap (mmol/L)	13.0 (10.0-17.2)	15.0 (11.3-19.4)	<0.001*
Calcium (mg/dL)	8.1 (7.5-8.7)	7.9 (7.2-8.5)	<0.001*
Creatinine (mg/dL)	4.4 (2.8-6.4)	3.0 (2.2-4.3)	<0.001*
Glucose (mg/dL)	150.3 ± 70.8	150.4 ± 69.5	0.957
Platelet count (×1000/µL)	184.0(119.0-255.5)	123.2(67.9-207.6)	<0.001*

Potassium (mmol/L)	4.5 (4.0-5.3)	4.5 (4.0-5.2)	0.113*
Sodium (mmol/L)	137.0 (134.0-140.5)	139.0 (135.0-142.5)	<0.001*
GCS score	14.0 (10.0-15.0)	9.0 (6.2-13.2)	<0.001*
MAP (mmHg)	76.0 (68.0-88.0)	69.7 (64.1-77.2)	<0.001*
HR (beats per minute)	87.0 (74.8-100.1)	94.0(80.1-106.4)	<0.001*
RR (breaths per minute)	19.4 (16.6-23.0)	22.2 (18.4-26.7)	<0.001*

Data are expressed as n (%) for categorical data and as mean ± standard deviation or median (interquartile range) for continuous data.

* Mann-Whitney U test

Table S5. Comparison of baseline characteristics of the patients requiring renal replacement therapy between the eICU and MIMIC datasets

Database	eICU	MIMIC	*P* value
Number of patients	2,283	1,129	
Death %	32.7%	42.9%	<0.001
**Demographics**			
Age, years	62.8 ± 14.6	63.0 ± 15.7	0.741
Male sex, %	58.4%	62.0%	0.047
Black race, %	13.2%	10.7%	<0.001
**Comorbidities, (%)**			
DM	14.6%	35.6%	<0.001
HTN	12.5%	52.4%	<0.001
CKD	16.6%	18.8%	0.125
CHF	16.9%	41.9%	<0.001
Malignancy	4.9%	10.4%	<0.001
LC	6.0%	19.3%	<0.001

Days of ICU stay before RRT initiation	1.1 (0.2-3.1)	1.5 (0.5-4.0)	<0.001*
Diuretics, %	12.0%	17.8%	<0.001
Vasopressor, %	36.9%	36.7%	0.914
**Laboratory variables, (mean)**			
BUN (mg/dL)	56.0 (36.0 -81.4)	59.7 (37.3-92.5)	0.001*
FiO2 (%)	44.5 (30.0-70.0)	40.8 (29.5-66.4)	<0.001*
HCO3 (mmol/L)	21.0 (16.8-24.5)	20.0 (16.5-23.8)	0.002*
Hgb (mg/dL)	9.4 (8.2-10.9)	9.9 (9.1-10.9)	<0.001*
O2 Sat (%)	96.6 (94.0-98.4)	95.0(89.0-97.0)	<0.001*
WBC count (x1000/μL)	15.8 ± 22.3	15.7 ± 27.8	0.873
Anion gap (mmol/L)	13.9 (10.5-18.0)	19.2 (16.0-23.0)	<0.001*
Calcium (mg/dL)	8.0 (7.4-8.6)	8.3 (7.7-9.0)	<0.001*
Creatinine (mg/dL)	4.6 ± 3.3	4.5 ± 2.9	0.354
Glucose (mg/dL)	133.5 (107.0-171.1)	126.7 (103.0-159.7)	<0.001*

Platelet count (x1000/µL)	167.0 (97.0-244.0)	151.1 (89.5-137.0)	0.056*
Potassium (mmol/L)	4.7 ± 1.1	4.7 ± 0.9	0.713
Sodium (mmol/L)	138.0 (134.0-141.0)	137.0 (133.8-140.5)	0.004*
GCS score	12.0 (8.0-15.0)	13.3 (7.2-15.0)	0.292*
MAP (mmHg)	76.5 ± 15.7	75.0 ± 13.2	0.006
HR (beats per minute)	89.9 ± 18.5	88.8 ± 18.1	0.114
RR (breaths per minute)	21.0 ± 5.6	20.8 ± 5.5	0.267

Data are expressed as n (%) for categorical data and as mean ± standard deviation or median (interquartile range) for continuous data.

* Mann-Whitney U test

Table S6. Pairwise *P* value of area under ROC curves (AUROCs) of prediction models using the Delong test in the eICU dataset.

AUROC	LR	XGBoost	RF	MLP
LR	1	0.58	0.48	0.08
XGBoost	0.58	1	0.81	0.95
RF	0.48	0.81	1	0.89
MLP	0.08	0.95	0.89	1

Table S7. Pairwise *P* value of area under ROC curves (AUROCs) of prediction models using the Delong test in the 20% pooled dataset.

AUROC	LR	XGBoost	RF	MLP
LR	1	0.68	0.76	<0.05
XGBoost	0.68	1	0.84	<0.05
RF	0.76	0.84	1	<0.05
MLP	<0.05	<0.05	<0.05	1

Table S8. Development of multivariable logistic regression model using stepwise variable selection in the training dataset.

Variable	Univariate Analysis		Multivariate Analysis	
	OR (95% CI)	P value	OR (95% CI)	P value
Female sex	0.93 (0.78-1.11)	0.42	0.82 (0.66-1.02)	0.079
Age	1.01 (1.01-1.02)	<0.001	1.03 (1.02-1.04)	<0.001
Vasopressor	2.87 (2.40-3.43)	<0.001	1.45 (1.15-1.83)	0.002
BUN	1.00 (1.00-1.00)	0.419	1.01 (1.00-1.01)	<0.001
FiO2	1.02 (1.01-1.02)	<0.001	1.02 (1.01-1.02)	<0.001
HCO3	0.95 (0.94-0.97)	<0.001	0.98 (0.96-1.01)	0.117
O2_sat	0.98 (0.97-0.99)	<0.001	0.98 (0.97-1.00)	0.02
WBC	1.02 (1.01-1.03)	<0.001	1.01 (1.00-1.02)	0.006
Anion Gap	1.05 (1.03-1.06)	<0.001	1.07 (1.05-1.10)	<0.001
Creatinine	0.81 (0.78-0.84)	<0.001	0.78 (0.73-0.83)	<0.001
Platelet	1.00 (0.99-1.00)	<0.001	1.00 (1.00-1.00)	<0.001
Malignancy	1.71 (1.23-2.39)	0.002	1.36 (0.91-2.05)	0.138
LC	3.16 (2.41-4.15)	<0.001	2.84 (2.01-4.05)	<0.001

GCS	0.87 (0.85-0.89)	<0.001	0.92 (0.89-0.94)	<0.001
HR	1.01 (1.01-1.02)	<0.001	1.01 (1.00-1.01)	0.065
MAP	0.97 (0.96-0.97)	<0.001	0.98 (0.98-0.99)	<0.001
RR	1.07 (1.06-1.09)	<0.001	1.03 (1.01-1.05)	0.007
Days before dialysis	1.05 (1.03-1.07)	<0.001	1.03 (1.00-1.05)	0.035
MV	3.18 (2.55-3.98)	<0.001	1.37 (1.02-1.86)	0.039

Figure S1. Calibration curves of all models using MIMIC dataset as training dataset.

Logistic Regression

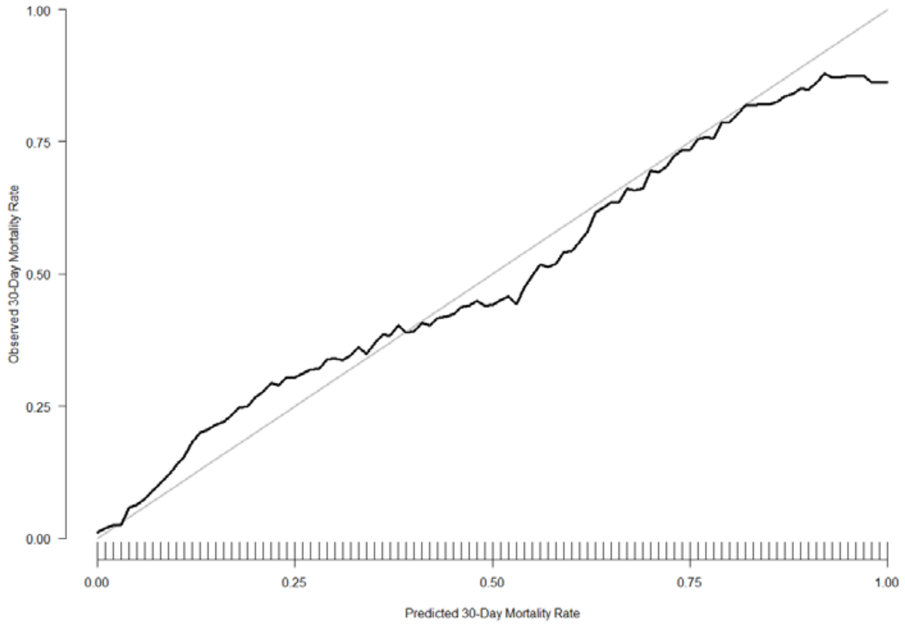

XGBoost

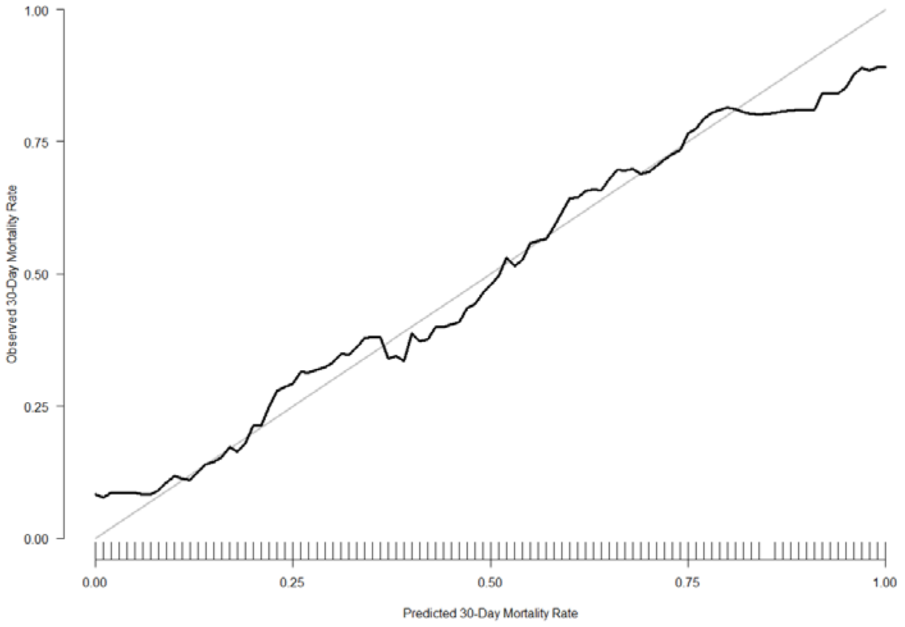

Random Forest

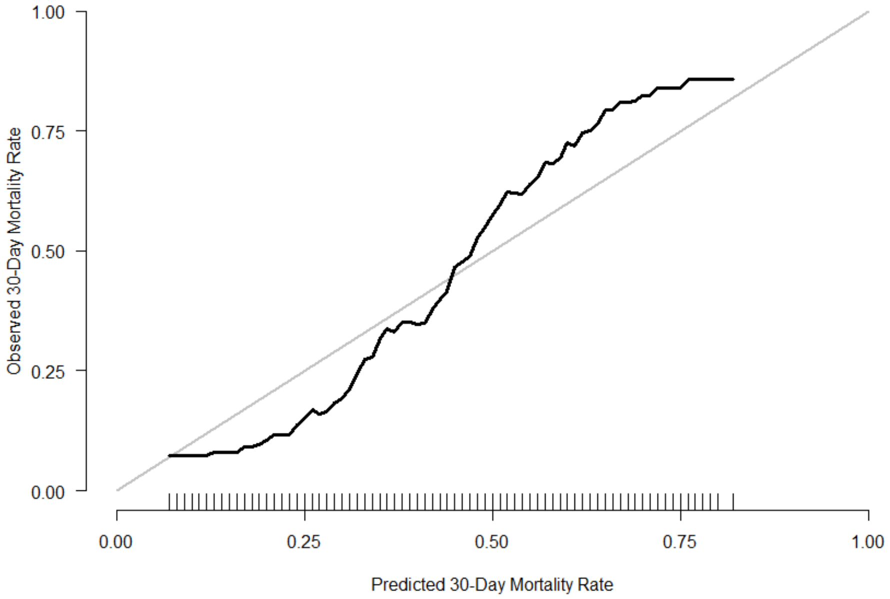

MLP

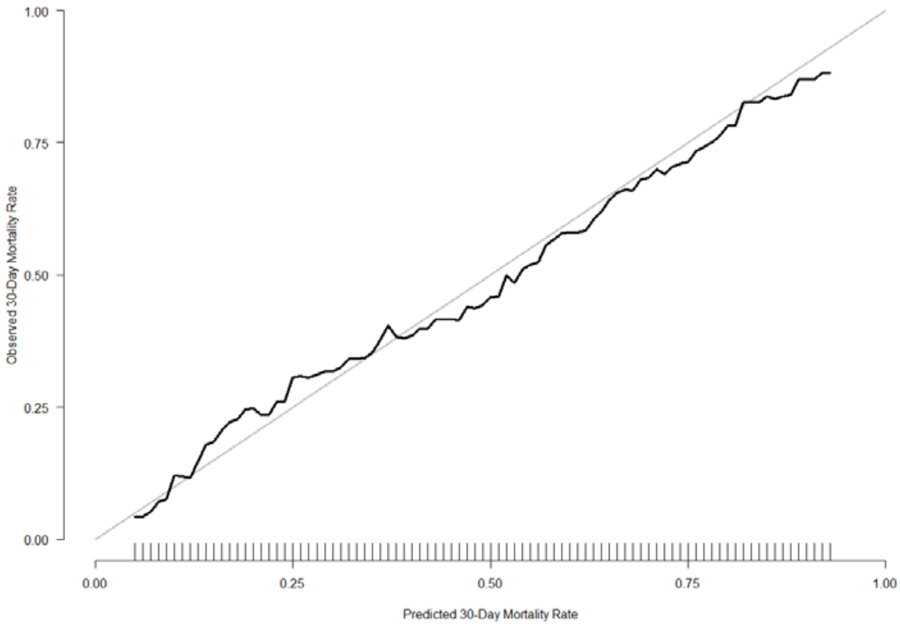

Figure S2. Calibration curves of all models using eICU dataset as testing dataset.

Logistic Regression

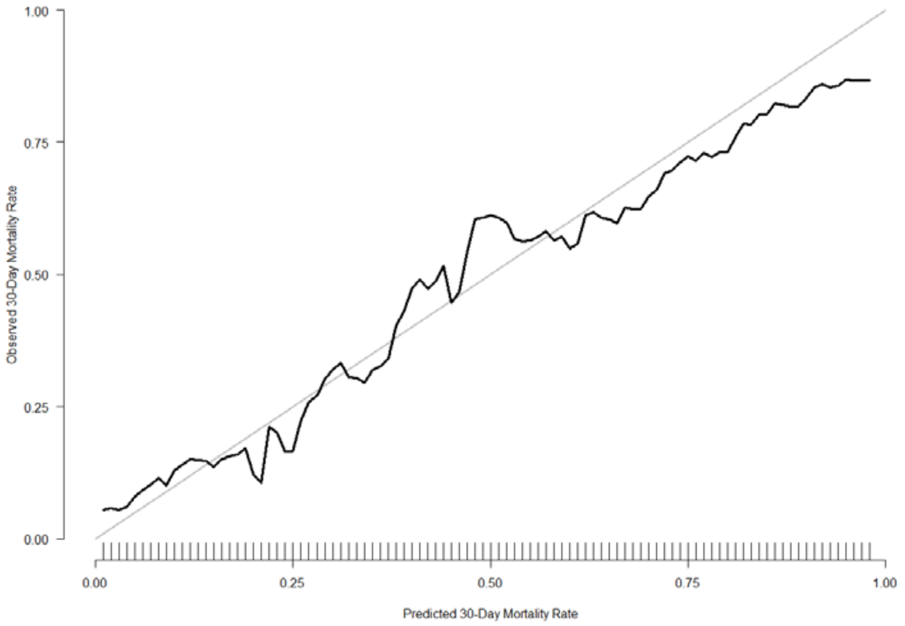

XGBoost

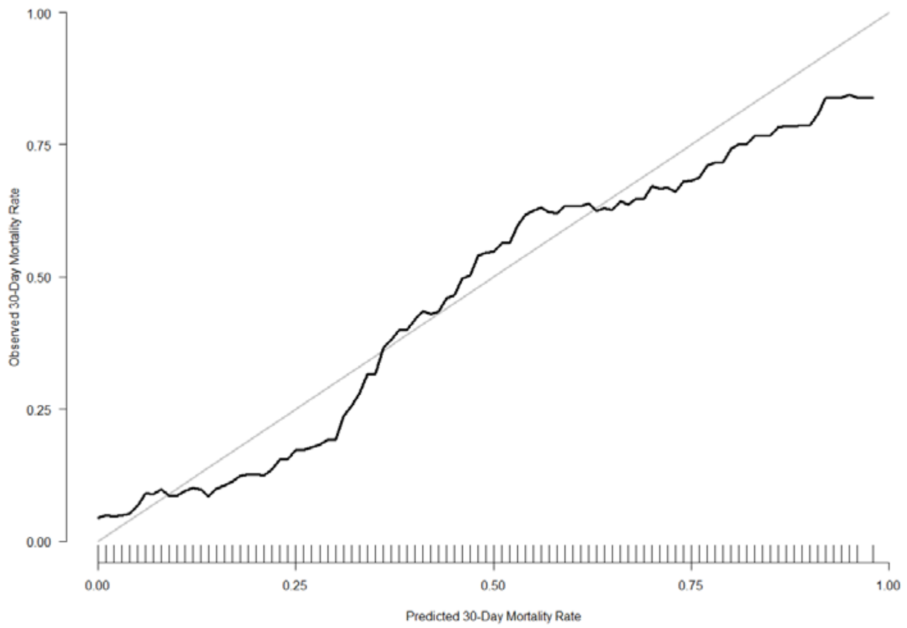

Random Forest

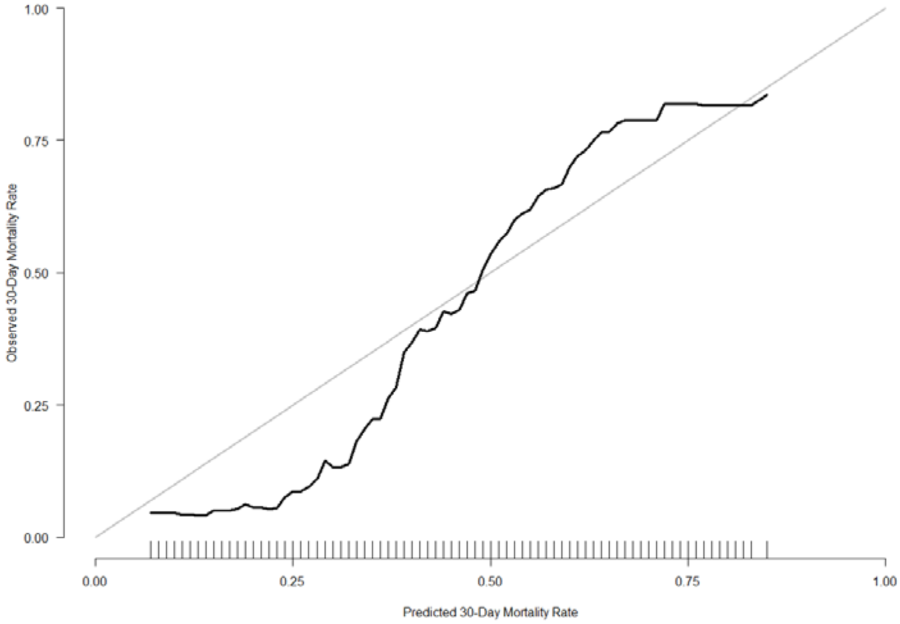

MLP

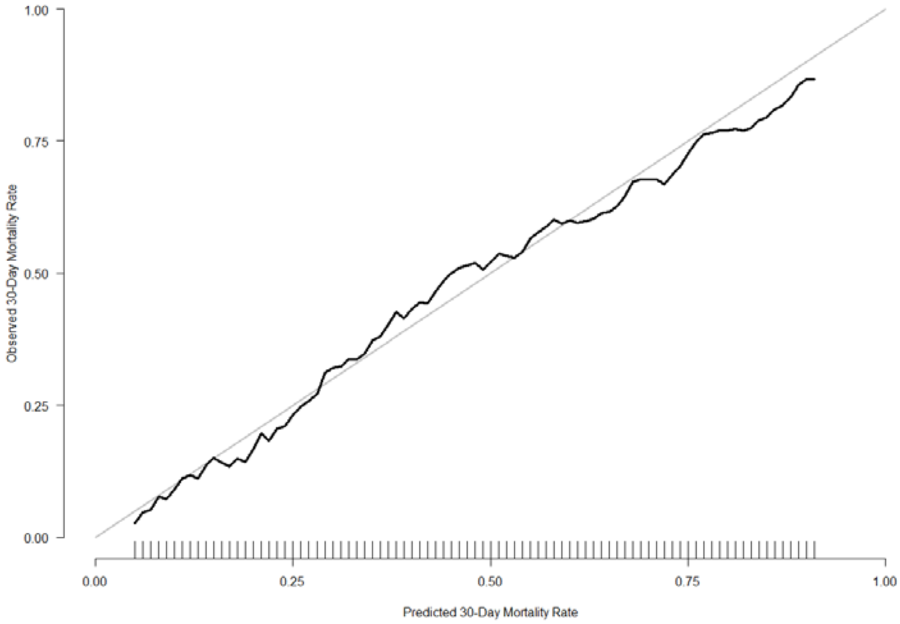

Figure S3. Calibration curves of all models using 80% pooled eICU and MIMIC dataset as training dataset.

Logistic Regression

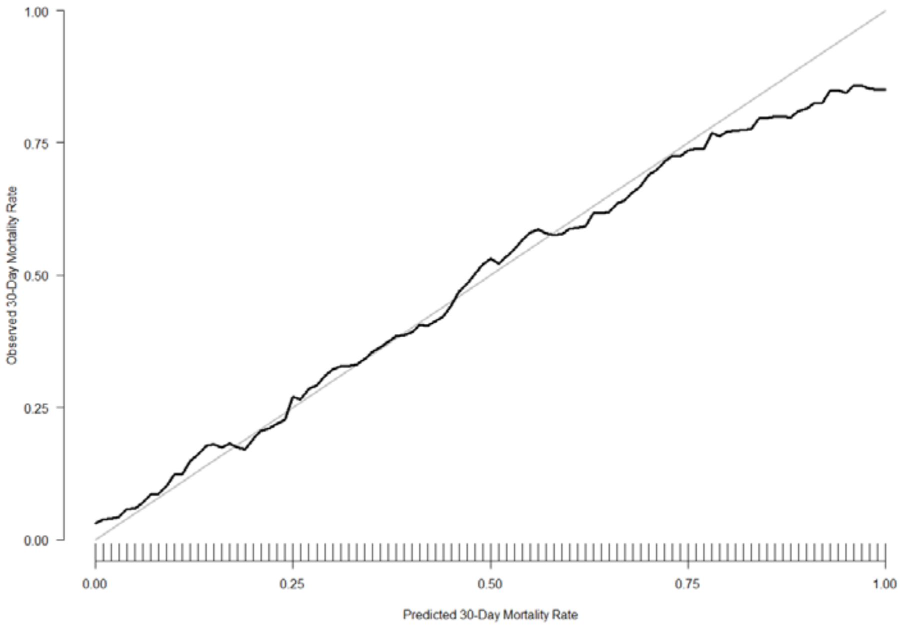

XGBoost

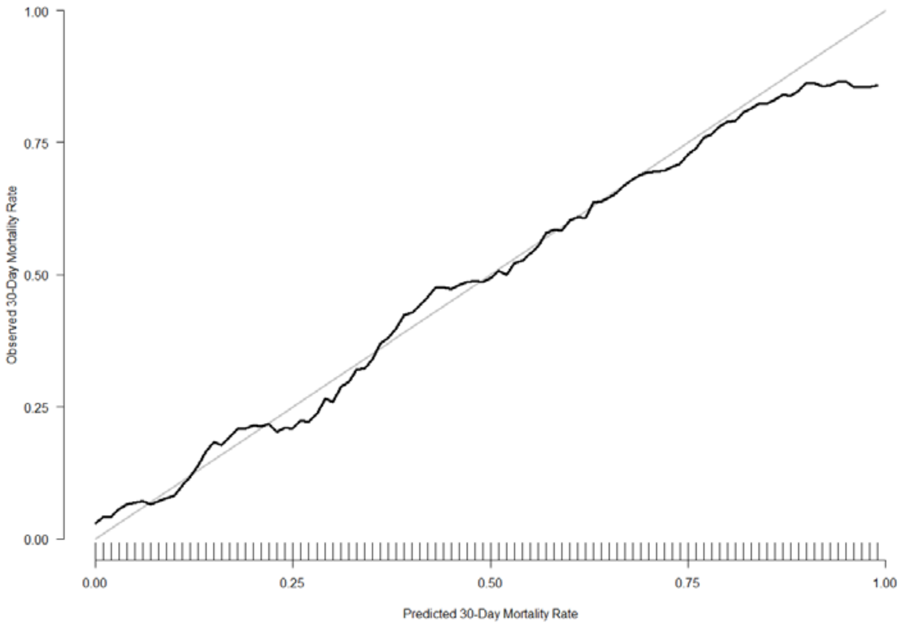

Random Forest

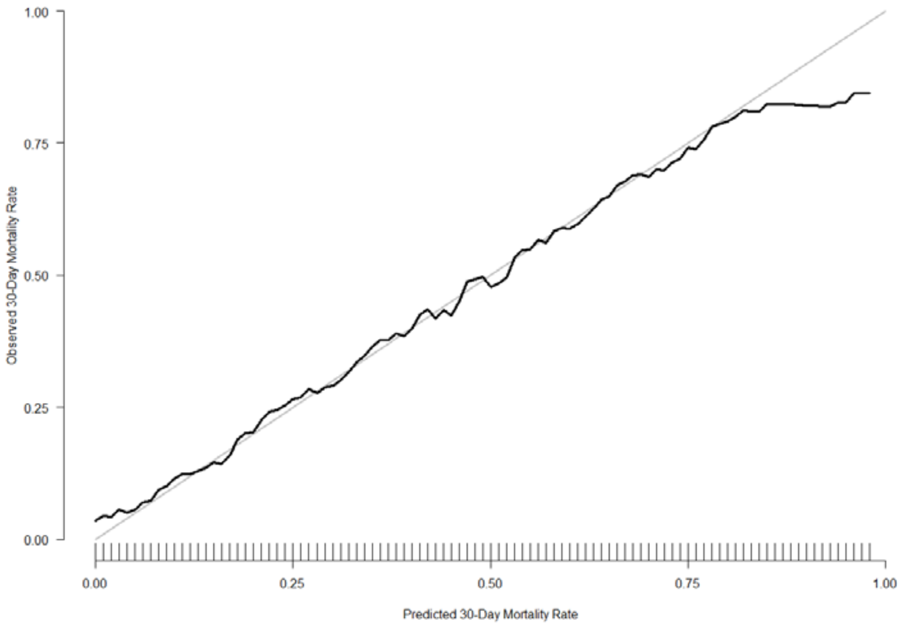

MLP

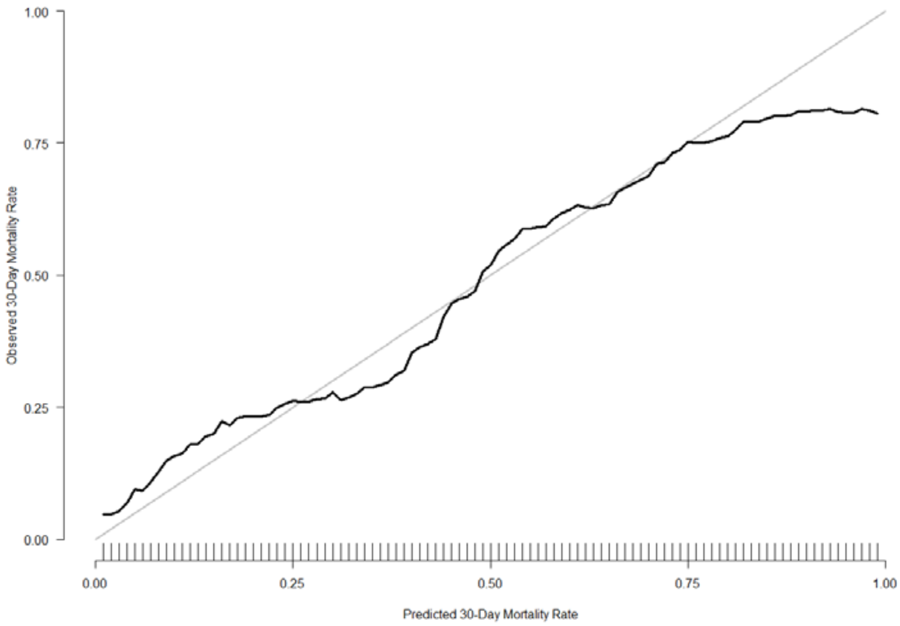

Figure S4. Calibration curves of all models using 20% pooled eICU and MIMIC dataset as testing dataset.

Logistic Regression

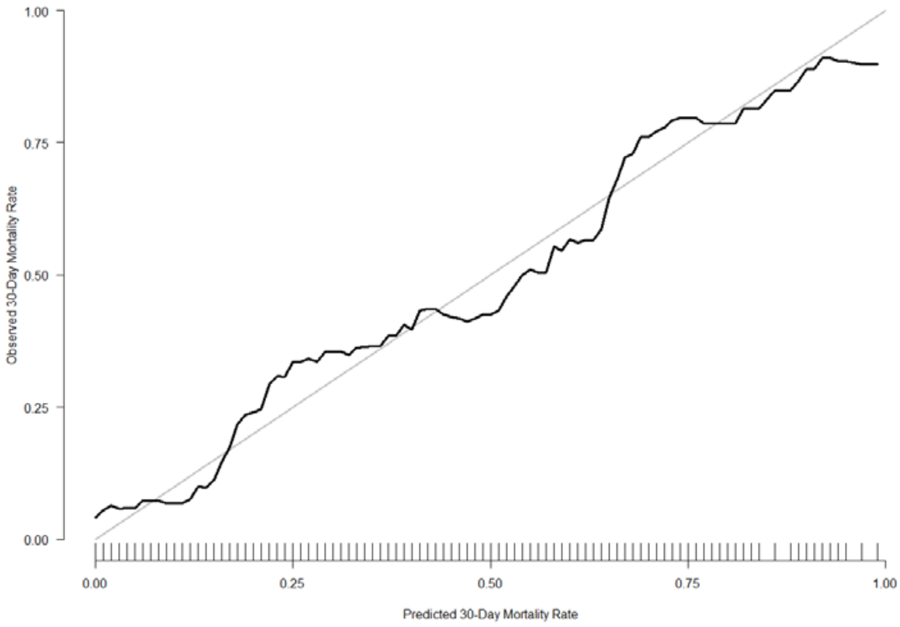

XGBoost

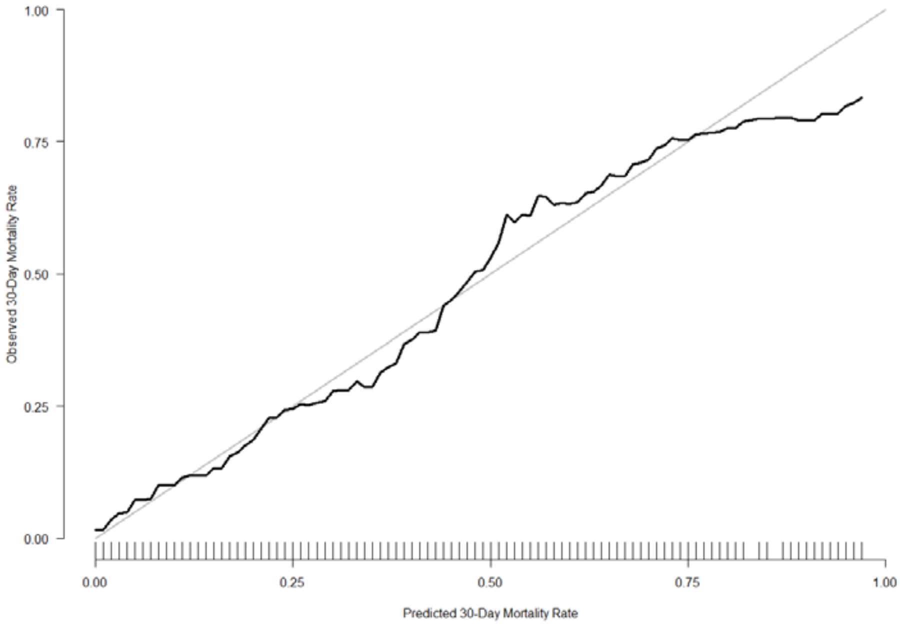

Random Forest

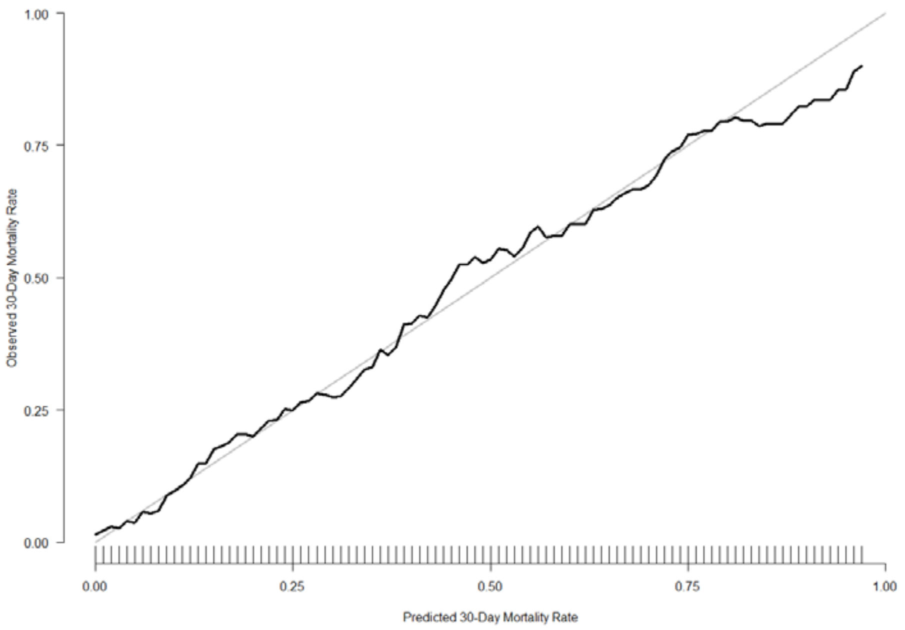

MLP

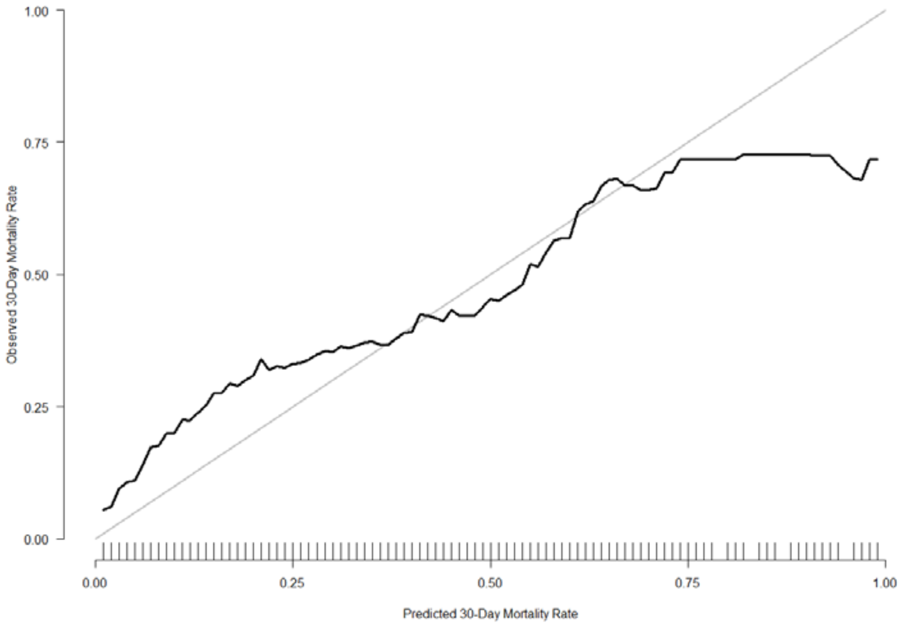

Figure S5. Principal component analysis (PCA) diagram reveals the similarity between death (1) and survival (0) group. The left figure is eICU dataset distribution and the right figure is MIMIC dataset distribution.

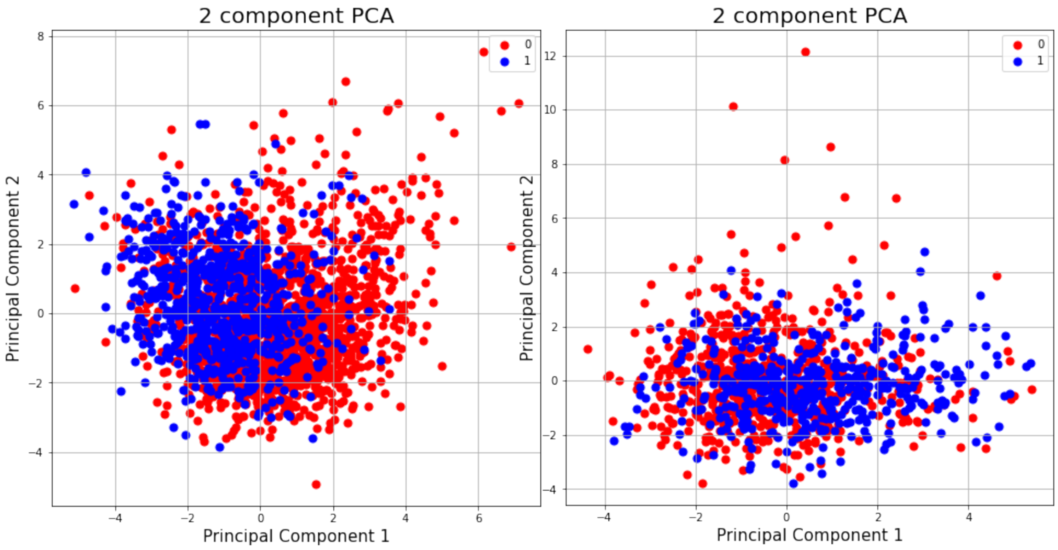

Supplement: Supplementary file 1 [file jcm-11-05289-s001.zip › jcm-1843070-supplementary.pdf]
